# Accelerated evolution and diversifying selection drove the adaptation of cetacean bone microstructure

**DOI:** 10.1186/s12862-019-1509-x

**Published:** 2019-10-24

**Authors:** Di Sun, Xuming Zhou, Zhenpeng Yu, Shixia Xu, Inge Seim, Guang Yang

**Affiliations:** 1 Jiangsu Key Laboratory for Biodiversity and Biotechnology, College of Life Sciences, Nanjing Normal University, Nanjing, 210023 China; 2 Key Laboratory of Animal Ecology and Conservation Biology, Chinese Academy of Sciences, Institute of Zoology, Beijing, China; 3 Integrative Biology Laboratory, College of Life Sciences, Nanjing Normal University, Nanjing, 210023 China; 4 Comparative and Endocrine Biology Laboratory, Translational Research Institute-Institute of Health and Biomedical Innovation, School of Biomedical Sciences, Queensland University of Technology, Brisbane, 4102 Australia

**Keywords:** Bone microstructure, Cetaceans, Adaptive evolution, Phylogenetic comparative analyses

## Abstract

**Background:**

The transition from land to sea by the ancestor of cetaceans approximately 50 million years ago was an incredible evolutionary event that led to a series of morphological, physiological, and behavioral adaptations. During this transition, bone microstructure evolved from the typical terrestrial form to the specialized structure found in modern cetaceans. While the bone microstructure of mammals has been documented before, investigations of its genetic basis lag behind. The increasing number of cetaceans with whole-genome sequences available may shed light on the mechanism underlying bone microstructure evolution as a result of land to water transitions.

**Results:**

Cetacean bone microstructure is consistent with their diverse ecological behaviors. Molecular evolution was assessed by correlating bone microstructure and gene substitution rates in terrestrial and aquatic species, and by detecting genes under positive selection along ancestral branches of cetaceans. We found that: 1) Genes involved in osteoclast function are under accelerated evolution in cetaceans, suggestive of important roles in bone remodeling during the adaptation to an aquatic environment; 2) Genes in the Wnt pathway critical for bone development and homeostasis show evidence of divergent evolution in cetaceans; 3) Several genes encoding bone collagens are under selective pressure in cetaceans.

**Conclusions:**

Our results suggest that evolutionary pressures have shaped the bone microstructure of cetaceans, to facilitate life in diverse aquatic environments.

## Background

A bony skeleton is vital to many adaptive phenotypes in vertebrates and represented a major leap in evolution [1]. The microstructure and organization in bone reflects the biomechanical constraints that organisms undergo and generally show a strong ecological signal [2–5]. Bone microstructure has been used to infer the habitat and locomotor mode of extinct taxa, as well as to assess the ecological, biomechanical, and phylogenetic significance of bone microstructure of aquatic amniote groups [2, 4, 6–8]. Cetaceans (whales, dolphins, and porpoises) are the most speciose order of marine mammals (~ 89 extant species in 14 families) and inhabit diverse habitats, including ocean basins and large riverine ecosystems [9]. Extant cetaceans exhibit osteological adaptations to an aquatic lifestyle, accompanied with complex buoyancy control systems [10–13]. Taken together, cetaceans represent a model group for the study of bone microstructure adaptations by ecological transition.

Extensive anatomical records on bone microstructure exist. The link between bone microanatomy (e.g. limbs, vertebrae, and ribs) and habitat has been studied by various investigators [8, 10, 11, 13–19]. In general, flying taxa (e.g. bats) exhibit a ‘simple’ bone microanatomy, with thin cortices and few trabeculae in the medullary region [20]. Terrestrial mammals usually display an intermediate cortical thickness compared to aquatic mammals [19]. The specialized bone microstructure in most extant whale exhibits a thin layer of compact cortex, lacks a medulla, and has been described as ‘osteoporotic-like’ but without bone mass decrease or any pathological connotation [2]. Other types of bone microstructure, such as non-pathological densification (osteosclerosis) and swelling (pachyostosis) with increased bone density, are observed in relatively inactive shallow water dwellers such as Sirenia and Archaeocetes [8, 10, 21]. These specializations have been attributed to hydrodynamic or hydrostatic control of buoyancy, as well as diving and swimming [10, 22, 23].

The bony skeleton of vertebrates is a dynamic and metabolically living organ constituted primarily of calcium-phosphate minerals and type I collagen. The growth of bones is sculpted by modeling and continuously renewed by remodeling [1]. The bone remodeling process, which occurs throughout a lifetime to maintain mineral homeostasis, involves timed expression of osteoclasts and osteoblasts to balance the bone matrix [24]. Multiple genes and/or pathways must have been involved in the genetic adaption of bone microstructure in aquatic mammals. However, unlike the relatively well documented morphological changes of bone microstructure in cetaceans, the underlying genetic basis has not been well addressed in the literature. Multiple cetacean genomes are now available, allowing this question to be answered. To examine the evolution of bone microstructure in cetaceans, we contrasted the bone microstructure of cetaceans to other mammals and identified associated gene-phenotype correlations, and assessed the selective pressure of bone-remodeling genes on the ancestral lineage of cetaceans.

## Methods

### Sample collection and bone microstructure measurements

**Fig. 1:**
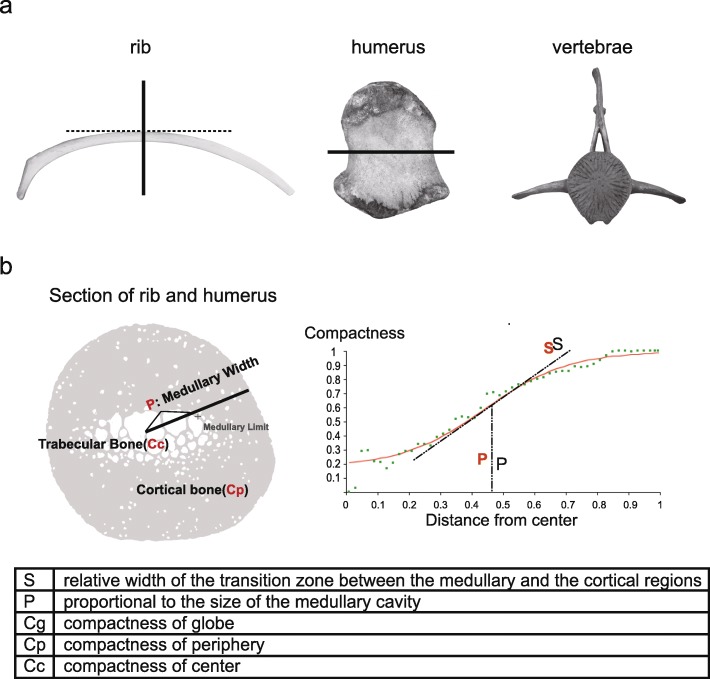
Characterization of bone microstructure. **a** Technical processing of the three kinds of bone, including long bone (rib and humerus) and vertebrae. Vertebrae data were obtained from [17], rib and humerus were sampled at the cross section at mid-length (solid line). Images of three bones were obtained from our laboratory. **b** Compactness profile indices for rib and humerus (*Cg*, *Cc*, *Cp*, *S* and *P*). Measured using Bone Profiler [25]. Each cross-section picture was converted to a binary image using Adobe Photoshop CS6

Anatomical data on bone microstructures (ribs 82 species, humeri of 14 species, and vertebrae of 50 species) (Additional file 1: Table S1–3) were obtained from two ways: (1) We collected and generated rib and humerus data from eight cetaceans and four adult terrestrial mammals in our lab – the false killer whale (*Pseudorca crassidens*), long-beaked common dolphin (*Delphinus capensis*), minke whale (*Balaenoptera acutorostrata scammony*), pantropical spotted dolphin (*Stenella attenuate*), Chinese white dolphin (*Sousa chinensis*), baiji (*Lipotes vexillifer*), finless porpoise (*Neophocaena asiaeorientalis*), common bottlenose dolphin (*Tursiops truncatus*), cow (*Bos taurus*), pig (*Sus scrofa*), sheep (*Ovis aries*), and dog (*Canis lupus familiaris*). Only dead stranding cetaceans for unknown reason in the wild was used in this study, and other mammal bone tissues were purchased in the market. No ethical approval was required. Bone sections were prepared as outlined by Canoville et al. and by Hayashi and colleagues [12, 19] (Fig. 1a). (2) To improve the sample size, sample images and information of more species was retrieved from previous various studies [2, 6, 12, 13, 19, 25].

We employed binary images of thin sections and assessed bone density using Bone Profiler [25], to obtain the variables *S* (relative width of the transition zone between the medullary and the cortical regions) and *P* (proportional to the size of the medullary cavity) for each section. The compactness of the center/periphery/whole of bone sections was calculated using Image-Pro Plus, to obtain the variables *Cg* (globe compactness, ranging from 0 ~ 1), *Cc* (compactness in the center of the section. *Cc* values > 0 usually indicates the presence of trabeculae in the center of a section), and *Cp* (compactness in the periphery of a section. *Cp* values < 1 indicates that the cortical region displays porosity). All of these variables represent the proportion of mineralized bone tissue occupying the total sectional area (Fig. 1b). The parameter *MD* is the maximal diameter of bone section that can be used as a proxy for body size in statistical analysis of rib and humerus microanatomical data [6, 19].

Measurements of vertebrae from 50 species were retrieved from Houssaye and colleagues [17]. Measurements where more than half of the values were missing were excluded from our analysis. We included nine variables in the subsequent analyses: *Cls* (global compactness of the centrum in longitudinal section), *CtsC* (centrum compactness in transverse section), *TNCL* (total number of cavities in longitudinal section), *NTCL* (number of trabeculae in the centrum longitudinal section), *AMCT* (absolute mean cortical thickness in transverse section), *RMCT* (relative mean cortical thickness in transverse section), *AMTT* (absolute mean trabecular thickness), *RMTT* (relative mean trabecular thickness) and *CL* (centrum length used as proxy of body size in statistical analysis of vertebrae microanatomical data).

### Statistical analysis of bone microanatomical data

Principal component analysis (PCA) was employed in order to reduce the dimensions of vertebrae indices. Vpc1 and Vpc2 (the first two principal components) explained 79% of the total variance (53 and 26%, respectively) of vertebrae compactness. Thus, *Vpc1* and *Vpc2* for vertebrae, and *S*, *P*, *Cc*, *Cp*, and *Cg* for rib variables were next used in phylogenetic generalized least squares (PGLS) multiple regression against body size variables (*CL* and *MD* for vertebrae and rib variables, respectively). Phylogenetic ANOVA analysis were employed to assess differences between habitat of bone variables for humerus, rib, and vertebrae using the ‘*phytools*’ package in R [26].

For the rib data set (*n* = 82), we further divided aquatic habitat species into shallow water/coastal water swimmers, and deep divers based on ecological behavior characteristics and diving depth data [27]. To assess bone histological parameters of 24 marine mammals we compared the *S*/*P*/*Cc*/*Cp*/*Cg* of ribs in six habitat categories in box plot and performed PGLS regression analysis of these variables against diving depth (in meters).

### Orthologous preparation and phylogenetic comparative analyses

**Fig. 2:**
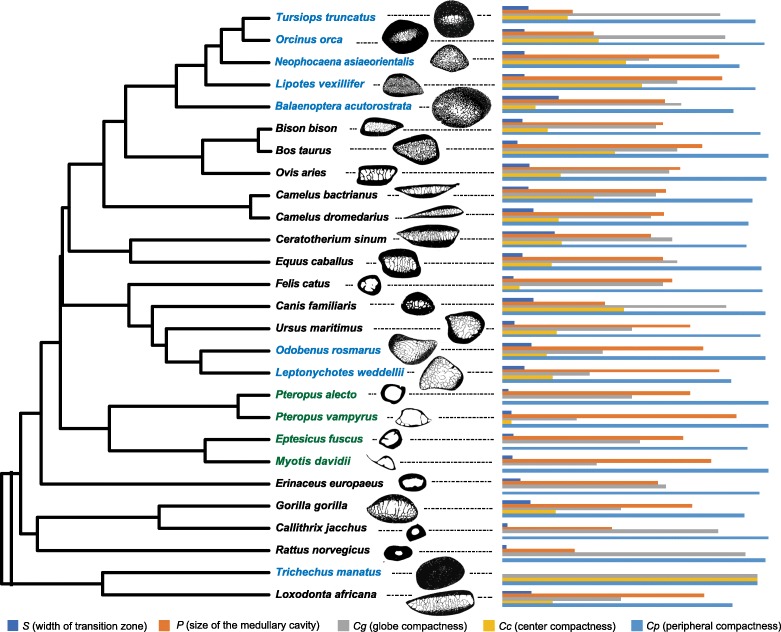
Phylogenetic tree of 27 species used in regression analyses. Representatives from marine, terrestrial, and flying mammals are in blue, black, and green, respectively. Binary cross section images of ribs with variation in five compactness indices were shown for each species. Bone section images were obtained from [19] and our lab: *Tursiops truncatus* (PL15–0145-28), *Orcinus orca* (AMNH 34261), *Neophocaena asiaeorientalis* (PL15–0145-21), *Lipotes vexillifer* (PL15–0145-18), *Balaenoptera acutorostrata* (PL15–0145-08), *Bison bison* (MHNL 50002450), *Bos taurus* (PL15–0145-33), *Ovis aries* (PL15–0145-39), *Camelus bactrianus* (MHNL 50002066), *Camelus dromedaries* (MHNL 50002063), *Ceratotherium sinum* (AMNH 51855), *Equus caballus* (MHNL 50002029); *Felis catus* (represent by *Felis silvestris p.c.* VB), *Canis familiaris* (PL15–0145-43), *Ursus maritimus* (p.c. VB), *Odobenus rosmarus* (MHNL 50001014), *Leptonychotes weddellii* (represent by *Cystophora cristata* AMNH 184659), *Pteropus Alecto* (represent by *Eidolon helvum* ZFMK no sp. number), *Pteropus vampyrus* (represent by *Pteropus giganteus* ZFMK 80.851), *Eptesicus fuscus* (represent by *Rousettus aegyptiacus* ZFMK 2001.004), *Myotis davidii* (represent by *Pipistrellus pipistrellus* ZFMK a), *Erinaceus europaeus* (p.c. VB), *Gorilla gorilla gorilla* (MHNL 50001762), *Callithrix jacchus* (ZFMK MAM_1983.0366), *Rattus norvegicus* (p.c. VB), *Trichechus manatus* (represent by *Dugong dugon* MHNL 50002521), *Loxodonta africana* (represent by *Elephas maximus* MHNL 50002671)

Of the species used in the quantitative analysis of bone microanatomy, 27 (or a related species in the same family or similar habitat or locomotion) had corresponding whole-genome sequences (Fig. 2). We generated three bone microstructure data sets: ribs for 27 species, humeri for 14 species, and vertebrae for 13 species (Additional file 1: Table S4). We next obtained genome sequences from NCBI and obtained 1:1 orthologous genes among them using OrthoMCL v2.0.9 [28]. Of 3621 single-copy orthologs, 348 were classified as bone function-associated genes based on GO terms (‘skeletal system development’, ‘ossification’, ‘bone remodeling’, ‘osteoblast proliferation’, ‘osteoclast differentiation’, and ‘osteoclast proliferation’), KEGG pathway names (‘osteoclast differentiation’), and a literature survey (key words ‘bone development’ and/or ‘osteoclast’) [29, 30]. Multiple alignments of orthologous sequences were generated using PRANK v150803 [31], followed by Gblocks v0.91b [32] and manual curation of alignments.

The ‘root-to-tip’ *d*_N_/*d*_S_, defined as the average value of accumulated *d*_N_/*d*_S_ extending from the last common ancestor of all mammals examined to the respective terminal branch, was estimated with PAML v4.4 [33] using a free-ratio model and parsed using custom Perl scripts. This measurement has been recognized as an index of selection which takes the entire evolutionary history of a lineage from a common ancestor into account and negates the issue of temporal effects on *d*_N_/*d*_S_ [34]. Regression between ‘root-to-tip’ *d*_N_/*d*_S_ and bone histological parameters was assessed using PGLS regression models under a phylogenetic framework across mammals (using the R package *‘caper’* v0.5.2) [35]. Topology and divergence date of a 27-species phylogenetic tree was obtained from the online resource TimeTree [36]. Briefly, PGLS employs a phylogenetic tree as input to assess the impact of phylogenetic non-independence between species. For every regression analysis, a quantitative measure of phylogenetic signal (Pagel’s lambda; λ) is calculated through maximum likelihood estimations. A λ value of 1, of or near 1, indicates that a variable is fully explained by evolutionary history and thus shows a strong phylogenetic signal [37, 38]. To obtain more stringent correlation *P* values, we further employed a two-step verification procedure [39]. On the basis of ‘*P value.all*’ from the regression analyses for all samples, the following two *P* values were calculated: 1) ‘*P value.robust*’ from PGLS repeated after excluding the sample with largest residual error; 2) ‘*P value.max*’ from the regression on the remaining species, to calculate the maximal *P* value after dropping one species.

### Estimating selective pressure

To identify genes that had been under selection, we set the ancestral branch of cetaceans as the foreground branch. Two models, the branch-site model [40] and clade model C [41], were implemented using *codeml* in PAML v4.4 [33]. The *P* value of each gene was computed using likelihood ratio tests (LRTs). The clade model C can detect evidence of divergent selective pressures acting across the cetacean clade as the foreground compared with the remaining species in the tree as the background. We set each model with three initial ω values (0.5, 1, and 1.5), to obtain the robust average ω, and compared this result with model M2a_ref (nearly neutral) via LRTs. Only genes with a unchangeable likelihood value for three initial ω values were considered interesting. To identify associations between genes, we implemented STRING (v10.5) functional analysis [42]. STRING integrates predicted and experimentally confirmed relationships between proteins that are likely to contribute to a common biological purpose.

## Results

### Ecological signals of bone microstructure across mammals

For three anatomical datasets (Additional file 1: Table S1–3), significant positive correlations were found between body size and the rib variables *Cc* (*P* = 3.9 × 10^− 9^, *r*^2^ = 0.35), *Cp* (*P* = 1.03 × 10^− 3^, *r*^2^ = 0.16), *S* (*P* = 2.15 × 10^− 10^, *r*^2^ = 0.39), and between body size and the vertebrae variable *Vpc1* (*P* = 2.2 × 10^− 16^, *r*^2^ = 0.76) (Additional file 2: Figure S1). We classified habitats into five categories (aquatic, amphibious, arboreal, terrestrial, and flying. For the humerus, only aquatic and terrestrial were considered). Phylogenetic ANOVA analysis showed that the aquatic group was significantly different from other groups in terms of *S*, *Cc*, and *Cp* (*P* < 0.01 in rib; *P* < 0.05 in humerus) **(**Additional file 2: Table S5**)**.

Of the 24 marine mammals examined in rib data set (Additional file 1: Table S1), deep divers (false killer whale, *Pseudorca crassidens*; narwhal, *Monodon monoceros*; Blainville’s beaked whale, *Mesoplodon densirostris*; long-finned pilot whale, *Globicephala melas*; and hooded seal, *Cystophora cristata*) had lower *Cp*, *Cc*, *S* and *Cg*, and higher *P* (Additional file 2: Figure S2 and S3). Additionally, diving depth (max/average) significantly correlated with *Cp* (max depth; *P* < 0.05, *r*^*2*^ = 0.13; average depth; *P* < 0.05, *r*^*2*^ = 0.13) and *Cg* (max depth; *P* < 0.05, *r*^*2*^ = 0.13; average depth; *P* < 0.05, *r*^*2*^ = 0.19) (Additional file 2: Figure S4), suggesting that deep-diving species have a lower bone density. For example, the hooded seal (*C. cristata*) and Blainville’s beaked whale (*M. densirostris*) exhibited low global compactness and numerous thin bone trabeculae.

### Detection of selective pressure and gene–phenotype association analysis

The branch-site model was used to identify positively selected genes in a 27-species data set with genome and corresponding bone microstructure measurements (see Additional file 1: Table S4 and Fig. 2). Nine genes (*COL1A2*, *COL3A1*, *FSHR*, *IFNAR1*, *MEPE*, *MITF*, *NFATC3*, *TEC*, and *TNFRSF1A*) showed evidence of strong positive selection (likelihood ratio tests, LRTs *P* < 0.05) in the common ancestor of cetaceans. A total of 14 genes (*SPARC*, *COL2A1*, *COL9A1*, *COL5A2*, *COL3A1*, *HES1*, *CTNNB1*, *FZD4*, *RUNX2*, *DVL3*, *TEC*, *FOSL1*, *STAT1*, and *LCP2*) showed significant positive selection in cetaceans but not in the outgroup taxa (foreground ω_2_ > 1.00 and background ω_1_ < 1.00, LRT *P* < 0.05). Interestingly, assessment for divergent evolution (clade model C) identified genes related to ‘Wnt signaling’ and ‘regulation of osteoblasts’ (Table 1 and see Additional file 3: Table S6–7).

**Table 1:** Summary of genes with a ‘root-to-tip’ *d*_N_/*d*_S_ significantly correlated with indices of three kinds of bone (rib, humerus and vertebrae) and under positive selection (PSG) or divergent selection (DSG) in cetaceans

Gene	Gene name	Gene function	Model	Ref
Wnt pathway				
*CTNNB1*	Catenin β1	Intracellular signaling protein of the Wnt–β-catenin pathway	DSG	[43, 44]
*FZD4*	Frizzled Class Receptor 4	Receptor for Wnt proteins	DSG	
*DVL3*	Dishevelled Segment Polarity Protein 3	Signal transduction protein in Wnt pathway	DSG	
Hormone				
*FSHR*	Follicle Stimulating Hormone Receptor	FSHR is located in osteoclasts, FSH stimulates osteoclastogenesis and bone resorption	PSG	[45]
Collagen protein				
*COL1A2*	Collagen Type I Alpha 2 Chain	Abundant and widespread: dermis, bone, tendon, ligament	PSG (rib)	[46]
*COL2A1*	Collagen Type II Alpha 1 Chain	Cartilage, vitreous	DSG	
*COL3A1*	Collagen Type III Alpha 1 Chain	Skin, blood vessels, intestine	PSG, DSG (rib)	
*COL5A2*	Collagen Type V Alpha 2 Chain	Bone, dermis, cornea, placenta	DSG (humerus)	
*COL9A1*	Collagen Type IX Alpha 1 Chain	Cartilage, cornea, vitreous	DSG	
*SPARC*	Osteonectin	Required for the collagen in bone to become calcified	DSG	[47, 48]
Osteoblast differentiation and function				
*HES1*	Hes Family BHLH Transcription Factor 1	Inhibiting osteoblast function and inducing bone resorption	DSG	[49]
*RUNX2*	Runt-related transcription factor 2	Transcription factor driving osteoblastogenesis	DSG	[50, 51]
*MEPE*	Matrix Extracellular Phosphoglycoprotein	Mineralization, phosphate regulation and osteogenesis.	PSG	[52]
*STAT1*	Signal Transducer and Activator Of Transcription 1	An important role in endochondral bone formation and chondrocyte differentiation	DSG	[53]
Osteoclast differentiation and function				
*TEC*	Tec Protein Tyrosine Kinase	Activated by RANKL and Indispensable for osteoclastogenesis	DSG, PSG (humerus)	[54]
*LCP2*	Lymphocyte Cytosolic Protein 2	Adaptor molecules in osteoclastogenesis	DSG (humerus)	
*FOSL1*	FOS Like 1, AP-1 Transcription Factor Subunit	Induces transcription of Fosl1 in osteoclast differentiation	DSG (humerus)	[55]
*IFNAR1*	Interferon Alpha and Beta Receptor Subunit 1	Regulating osteoclast differentiation and bone resorption	PSG	[56]
*MITF*	Melanogenesis Associated Transcription Factor	Nuclear activity of osteoclast	PSG (humerus)	[57]
*TNFRSF1A*	TNF Receptor Superfamily Member 1A	TNF-α inhibit osteoblast differentiation and active osteoclastogenesis through TNFRSF1A	PSG (rib)	[58]

**Fig. 3:**
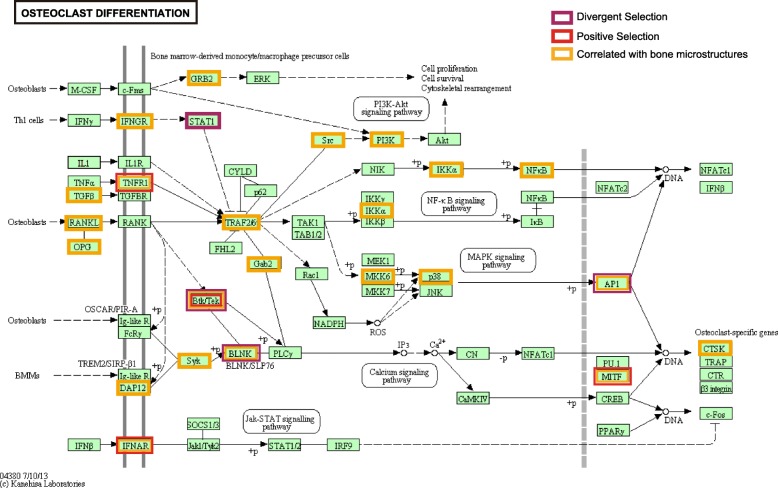
Mapping of genes with ‘root-to-tip’ *d*_N_/*d*_S_ significantly correlated with bone microstructures or under positive and divergent selections to the KEGG osteoclast differentiation pathway

**Fig. 4:**
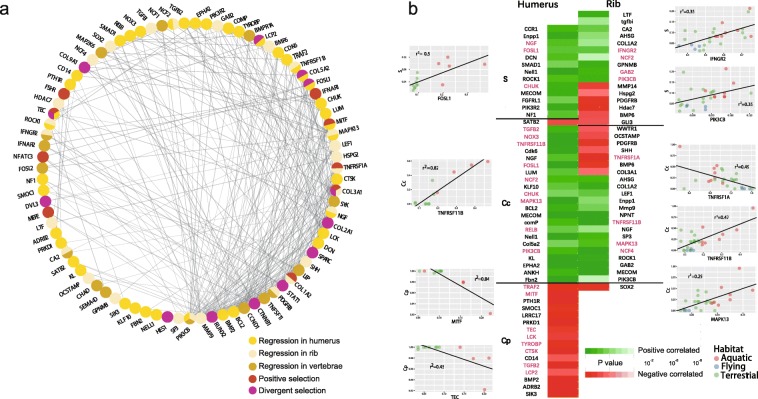
Overview of 83 genes with gene substitution rates correlating with bone microstructures. **a.** Protein–protein interaction network generated using STRING [42]. Nodes represent protein-coding genes correlating with bone variables (regression analysis, vs. ‘root-to-tip’ *d*_N_/*d*_S_). Lines between nodes indicate inferred or experimentally demonstrated biological associations. Humerus, rib, and vertebrae are indicated in yellow, beige, and brown respectively. Genes also under positive or divergent selection are indicated in maroon and purple, respectively. **b.** Heat map of genes correlating (green, positive; red, negative) with bone variables (regression analysis, vs. ‘root-to-tip’ *d*_N_/*d*_S_). Genes in the osteoclast differentiation pathway are highlighted in pink. For selected genes, a plot of bone variable (vertical axis; please see main text for details) and substitution rate (‘root-to-tip’ dN/dS) is shown; with salmon, blue, and green indicating species in an aquatic, flying, and terrestrial habitat, respectively

A comparison of ‘root-to-tip’ *d*_N_/*d*_S_ values **(**see Additional file 1: Table S4) and bone measurements from the ribs (27 species), humeri (14 species), and vertebrae (13 species) revealed 83 genes with substitution rates correlating with bone variables (see Additional file 4: Table S8–10). Since body size correlated positively with several variables (*S*, *Cc*, and *Cp* for rib; *Vpc1* for vertebrae) (Additional file 2: Figure S1), variable residuals were computed to account for body size influence on regression and employed in a new round of regression analysis. For the variables *Cc* and *Cp*, 83% of genes were significant or near significant (*P* value.all for residual < 0.1), while one gene was detected near significant each for the variables *S* (*S* ~ *NCF2*: *P* value.all for residual = 0.08) and *Vpc1* (*vpc1*~*SYK*: *P* value.all for residual = 0.083) (Additional file 4: Table S8). Of the 83 genes, 27 genes are associated with the KEGG pathway ‘Osteoclast differentiation’ (*P* < 0.1) (Fig. 3) and six were with ‘Phagosome’ (*P* < 0.01) (see Additional file 5: Table S11–12). Moreover, six genes (*PIK3CB*, *NGF*, *MAPK13*, *TNFRSF11B*, *NCF2*, and *IFNGR2*) associated with the osteoclast differentiation pathway showed a significant correlation with two out of three bone types (Fig. 4a). Four genes (*MITF*, *TNFRSF1A*, *TEC*, and *IFNAR1*) in the osteoclast differentiation pathway were identified by the branch site model, suggesting that they have undergone positive selection in cetaceans. Additionally, clade model C revealed that four genes (*STAT1*, *TEC*, *LCP2*, and *FOSL1*) have been subject to divergent selection pressures in cetaceans compared to other taxa (Fig. 3 and 4a; Table 1). With the exception of *TNFRSF1A* (TNF receptor superfamily member 1A), 26 genes enriched for the osteoclast differentiation pathway showed a negative correlation between ‘root-to-tip’ *d*_N_/*d*_S_ and *Cp* and a positive correlation between ‘root-to-tip’ *d*_N_/*d*_S_ and bone indices *Cc* and *S*, (Fig. 4b). Thus, genes associated with osteoclast function have evolved rapidly in aquatic mammals.

The association of ‘root-to-tip’ *d*_N_/*d*_S_ with bone mass parameters and the selective pressure analysis both identified several genes which encode collagen proteins (Table 1 and Fig. 4a), essential components of bone and cartilage and required for endochondral ossification. For example, genes encoding type I and III collagens (*COL1A2* and *COL3A1*) were under positive selection in the ancestral lineage of cetaceans. Although our two-step verification procedure (see [39]) revealed a relationship between S and Cc vs *d*_N_/*d*_S_ of *COL1A2* (*Cc*: *P* value.all = 0.002, *P* value.robust = 0.001, *P* value.max = 0.002; *S*: *P* value.all = 0.004, *P* value.robust = 0.007, *P* value.max = 0.049), this was not significant in regression analyses where variable residuals were employed to account for body size (*Cc*: *P* = 0.305; *S*: *P* = 0.238). Furthermore, the divergent selection scan showed that type II, V, and IX collagens (*COL2A1*, *COL5A2* and *COL9A1*) and osteonectin (*SPARC*) were also under positive selection in extant cetaceans.

## Discussion

### Bone microstructure in cetaceans with diverse ecological behaviors

Anatomical records from marine mammals (16 cetaceans, seven pinnipeds, and the sea otter) allowed us to address the great diversity of bone microstructure in marine mammals, especially for the fully aquatic cetaceans. Combining qualitative data and statistical analyses, we reveal that whole bone volume (*Cg*) does not scale substantially with habitat or body size. Mammals with a larger body size tend to have a more complex organization, with an increased transition zone (*S*) between the cortical bone and the medullary cavity, as well as numerous bone trabeculae occupying the medullary region (also called cancellous bone; *Cc*). For animals from different categories of habitats, the compactness of cortical bone (*Cp*) is relatively low in aquatic mammals, especially in deep-diving whales. Cortical bone is dense and strong enough to provide both support and protection for most mammals. However, for cetaceans, who harbor a more porous bone structure, it seems that the trabecular bone tends to invade into the cortical bone – as evidenced by higher *S* and *Cc* values. It is generally accepted that the trabecular bone is more responsive to, and malleable for, variations in magnitude and direction of load throughout life compared to cortical bone [59]. Therefore, this pattern of bone microstructure may be an adaptation for the aquatic lifestyle of cetaceans.

The inner bone structure within cetaceans is variable, especially in the case of the ribs. It has been assumed that ribs contribute significantly to the mass and inertia of the body in these “limbless” mammals [19]. Compared to most terrestrial mammals, which have a fully compact cortical bone, cetaceans display porosity (lower *Cp*). Some cetaceans in the Delphinidae and Monodontidae families exhibit unexpectedly thick cortices, with a certain extent of porosity and thicker bone trabeculae in the medullary region. This could be because an increased bone density supports feats such as the acrobatics and fast swimming speed of dolphins and feeding in deep divers [60, 61]. Another interesting observation is that species which reside in shallow freshwater, such as baiji (*Lipotes vexillifer*) and boto (*Inia geoffrensis*)*,* have a spongy section with numerous thin bone trabeculae and a low global compactness (*Cg*). Although the function of this modification is unknown, it may support relatively lower buoyancy in a freshwater habitat.

### Accelerated evolution of osteoclast differentiation-related genes in cetaceans

Previous studies suggested that down- and up-regulation of osteoclast activity was central to bone microstructure of tetrapods returning to an aquatic environment [10]. Our results reveal a positive correlation to *S* or *Cc* and negative correlation to *Cp* by genes in the osteoclast differentiation pathway. Since aquatic mammals had a relatively higher *S* and *Cc*, and a lower *Cp*, genes with higher ‘root-to-tip’ *d*_N_/*d*_S_ values in cetaceans could reflect accelerated evolution of osteoclastogenesis.

Most of the positively selected genes (e.g., *TEC*, *LCP2*, *TRAF2*, *MITF*, *CTSK*, *LCK*, and *GRB2*) in the ancestral branch of cetaceans are genes with ‘root-to-tip’ *d*_N_/*d*_S_ that negatively correlate with *Cp*. These genes encode intracellular signaling cascade proteins involved in osteoclast differentiation (see Fig. 4b and Table 1**)**. Mutations in, or the lack of any of these, genes would cause severe osteopetrosis [54, 57, 62–64]. The genes (*IFNAR1*, *TNFRSF1A*, *TNFRSF11B*, *TNFSF11*, and *TGFB2*) in the osteoclast differentiation pathway are ligands or receptors that trigger the differentiation process of osteoclasts **(**Fig. 3 and Table 1**)**. Two genes have a negative impact on osteoclast differentiation. The ‘root-to-tip’ *d*_N_/*d*_S_ of *TNFRSF11B* (osteoprotegerin; also known as *OPG*) positively correlated with *Cc* in both *rib* (*P value.robust* < 3.89 × 10^− 06^, *r*^*2*^ = 0.91) and humerus (*P value.robust* < 2.10 × 10^− 06^, *r*^*2*^ = 0.6), while *TNFSF11* (receptor activator of nuclear factor kappa B ligand; also known as *RANKL*) negatively correlated to *Vpc2* in vertebrae (*P value.robust* < 1.61 × 10^− 04^, *r*^*2*^ = 0.75). In GWAS meta-analyses these genes were associated with volumetric bone mineral density, suggesting that the *RANK-RANKL-OPG* axis affects the skeleton – at least in part by influencing the density of cortical bone [65]. Another positively selected gene in the cetacean lineage of interest is *TNFRSF1A* (also known as *TNFR1*), the only gene with a ‘root-to-tip’ *d*_N_/*d*_S_ negatively correlated with *Cc* (*P value.robust* = 3.93 × 10^− 05^, *r*^2^ = 0.49). *TNFRSF1A* is the receptor of TNF-α and modulates immune and inflammatory processes, as well as bone homeostasis [58]. Cross-talk between interferons and other cytokines in bone remodeling have recently received greater attention. For example, one of the IFN-β receptor components in the type I interferon system is *IFNAR1* **(**Table 1**)**. *Ifnar1*^−/−^ mice have markedly reduced trabecular bone mass, a key feature of osteoporosis, suggesting that the gene plays a critical role in osteoclastogenesis [56]. We hypothesize that bone homeostasis in aquatic mammals is maintained by the *RANK*-*RANKL*-*OPG* axis acting in concert with cytokines.

In summary, we have identified a correlation between accelerated changes of osteoclast-associated genes and increased trabeculae in cetaceans. This correlation could reflect increased bone resorption. Further analyses of selective pressure suggest that several genes related to osteoclast differentiation underwent accelerated change due to positive selection, likely to allow bone microstructure specialization in cetaceans.

### Divergent selection of bone formation genes between cetaceans and other mammals

Genes associated with ‘canonical Wnt signaling’ and ‘regulation of osteoblasts’ were shown to evolve through divergent selection in cetaceans compared to other taxa (Table 1). It is well known that Wnt signaling plays a pivotal role in skeletal homeostasis by regulating bone formation and bone resorption by osteoblasts and osteoclasts [43]. Genes associated with the Wnt intracellular signaling (*CTNNB1*, *FZD4*, and *DVL3*) were under divergent selection in cetaceans. Other genes under divergent selection encode transcription factors. This includes *RUNX2* which is essential for the maturation of osteoblasts [50]. Knockout of *RUNX2* in mice results in a complete lack of bone formation and arrested osteoblast differentiation [66]. Moreover, studies of mouse models with knockout or transgenic Wnt pathway components have demonstrated that this signaling pathway regulates most aspects of osteoblast physiology, including bone matrix formation/mineralization, osteoclastogenesis, and bone resorption [43, 44]. Thus, significant divergent selection of these genes between cetaceans and other mammals suggests that they played crucial roles in driving the bone development and formation in response to aquatic adaptations.

### Positive selection of collagen genes in cetaceans

The matrix of bone mainly contains collagen fibers and mineral deposits. Type I collagen in bone and type II collagen in cartilage provide structural integrity and account for mechanical strength [67]. Osteogenesis imperfecta, a severe genetic disorder manifested by increased bone fragility and low bone mass, is included in the diseases associated with *COL1A2* [46]. Alteration of collagen structural properties by missense mutations in *COL1A1* and *COL1A2* reduce the mechanical properties of bone [68]. Moreover, cetacean *COL1A2* harbors unique substitutions that may modify the collagen triple helix [69]. Nevertheless, the fact that *COL1A2* and several other genes mentioned before were not statistical significant after correcting for body size residuals. Possible reasons may include sample size or ecological/statistical implications for bone sample feature, e.g. only one specific region of the rib series were sampled or intraspecific microanatomical variability, which were mentioned in Canoville et al. (2016) [19]. Type III collagen (*COL3A1*) showed a negative correlation between evolutionary rate and Cc in cetaceans, as well as positive selection and divergent selection in the ancestral lineage of cetaceans. *COL3A1* is highly expressed during embryonic skeletal development and expressed by osteoblasts in mature bone. Both in vivo and in vitro experiments have shown that COL3A1 plays an important role in the development of trabecular bone through its effects on osteoblast differentiation [70]. Loss of *COL3A1* function is associated with Ehlers-Danlos syndrome (EDS), where skeletal manifestations include a distinctive facial appearance, scoliosis, and osteoporosis [71]. Cartilaginous collagen fibrils are represented by collagens type II, IX, and XI and endochondral ossification is gradually replaced by a bone matrix composed of type I and type II collagens [72]. Together with divergently selected osteonectin (*SPARC*), a gene which encodes a protein that bind to ECM proteins such as type I, III, IV, and V collagens to promote bone mineralization [47, 73], we speculate that bone collagen are likely to have contributed significantly to the specialization of cetaceans bone microstructure.

## Conclusions

Along with the transition of cetaceans from land to sea, changes in habitats and ecological behaviors in cetaceans resulted in diverse patterns of bone microstructures. In the present study, we found that genes involved in osteoclast differentiation experienced accelerated evolution in cetaceans. In addition, Wnt signaling and osteoblastogenesis associated with bone development were found to be under divergent evolution in cetaceans. We also detected positive selection of collagen proteins in cetaceans. These findings provide new insights into the nexus between genes and the secondary adaptation of terrestrial mammals to aquatic life.

## Supplementary information

**Additional file 1:** Table **S1-3.** Datasets of rib, humerus and vertebrae compiled in the present study, respectively. Containing the list of species sampled, the collection number, the habitat, and the bone histological parameters measured for each species. **Table S4.** List of ‘root-to-tip’ *d*_N_/*d*_S_ values of each species for 348 bone function-associated genes.

**Additional file 2:** **Table S5.** Data from phylogenetically informed ANOVA. Summary of pairwise comparison results of bone quantitative indices in different habitats using the *phytools* package in R. a. data from rib. b. data from humerus. **Figure S1.** Linear regressions result of *MD* and bone parameter (*S*, *Cc*, *Cp*) from rib; Linear regression result of *CL* and *Vpc1* from vertebrae. **Figure S2.** A phylogeny of 24 marine mammals with corresponding bone section and the max diving depth for each species. The section images are *Tursiops truncatus* PL15-0145-28, *Delphinus capensis* PL15-0145-07, *Stenella attenuata* PL15-0145-14, *Sousa chinensis* PL15-0145-16, *Globicephala melas* AMNH 215271, *Pseudorca crassidens* PL15-0145-03, *Lissodelphis borealis* AMNH 31422, *Orcinus orca* AMNH 34261, *Neophocaena asiaeorientalis* PL15-0145-21, *Phocoena phocoena* MHNL 50001046, *Delphinapterus leucas* AMNH 34936, *Monachus monachus* MHNL 50001018, *Inia goeffrensis* AMNH 209101, *Lipotes vexillifer* PL15-0145-18, *Mesoplodon densirostris* AMNH 139931, *Balaenoptera acutorostrata* *scammoni* PL15-0145-08, *Zalophus californianus* AMNH 63946, *Eumetopias jubatus* AMNH 38400, *Arctocephalus pusillus* AMNH 81701, *Odobenus rosmarus* MHNL 50001014, *Cystophora cristata *AMNH 184659, *Phoca vitulina* MHNL 50001020, *Monachus monachus* MHNL 50001018, *Enhydra lutris* MHNL 50001023. **Figure S3.** Box plot of six habitat categories for five bone indices from ribs. **Figure S4.** The linear regressions of max/average diving depth with Cp and Cg for 24 aquatic mammals.

**Additional file 3:** **Table S6.** List of positively selected genes (PSGs) and divergently selected genes (DSGs) in cetaceans identified from 348 bone function-associated genes. **Table S7.** List of divergently selected genes (DSGs) in cetaceans identified from 348 bone function-associated genes.

**Additional file 4:** **Table 8-10.** Result of regression analyses between ‘root-to-tip’ *d*_N_/*d*_S_ and bone histological parameters for rib, humerus and vertebrae, respectively.

**Additional file 5:** **Table S11.** Enrichment pathways for bone functional genes which ‘root-to-tip’ *d*_N_/*d*_S_ significantly correlated with bone histological parameters. **Table S12.** Enrichment GO categories for bone functional genes which ‘root-to-tip’ *d*_N_/*d*_S_ significantly correlated with bone histological parameters.

## Data Availability

The data generated and analyzed during this study are included in this article and its additional files 1-5.

## Abbreviations

AMNH: American Museum of Natural History, New York, NY, USA
GO: Gene Ontology
JRHRVC: uncatalogued research collection of John R. Hutchinson at The Royal Veterinary College, Hatfield, UK
MHNL: Musée des Confluences, Centre de conservation et d’étude des collections, 13A rue. Bancel, 69,007 Lyon, France
MNHN: Museum National d’Histoire Naturelle, Paris, France
OLS: ordinary least squares regression models
p.c. VB: Personal collection of V. de Buffrénil
PGLS: phylogenetic generalized least squares. Institutional abbreviations
ZFMK: Zoological Research Museum Alexander Koenig, Bonn, Germany
