# Supplementary material for: Accelerated evolution and diversifying selection drove the adaptation of cetacean bone microstructure
Source: BMC Evol Biol. 2019 Oct 24;19:194. doi: 10.1186/s12862-019-1509-x (PMC6813995; doi:10.1186/s12862-019-1509-x)
Supplement: Supplementary file 2 — Additional file 2: Table S5. Data from phylogenetically informed ANOVA. Summary of pairwise comparison results of bone quantitative indices in different habitats using the phytools package in R. a. data from rib. b. data from humerus. Figure S1. Linear regressions result of MD and bone parameter (S, Cc, Cp) from rib; Linear regression result of CL and Vpc1 from vertebrae. Figure S2. A phylogeny of 24 marine mammals with corresponding bone section and the max diving depth for each species. The section images are Tursiops truncatus PL15-0145-28, Delphinus capensis PL15-0145-07, Stenella attenuata PL15-0145-14, Sousa chinensis PL15-0145-16, Globicephala melas AMNH 215271, Pseudorca crassidens PL15-0145-03, Lissodelphis borealis AMNH 31422, Orcinus orca AMNH 34261, Neophocaena asiaeorientalis PL15-0145-21, Phocoena phocoena MHNL 50001046, Delphinapterus leucas AMNH 34936, Monachus monachus MHNL 50001018, Inia goeffrensis AMNH 209101, Lipotes vexillifer PL15-0145-18, Mesoplodon densirostris AMNH 139931, Balaenoptera acutorostrata scammoni PL15-0145-08, Zalophus californianus AMNH 63946, Eumetopias jubatus AMNH 38400, Arctocephalus pusillus AMNH 81701, Odobenus rosmarus MHNL 50001014, Cystophora cristata AMNH 184659, Phoca vitulina MHNL 50001020, Monachus monachus MHNL 50001018, Enhydra lutris MHNL 50001023. Figure S3. Box plot of six habitat categories for five bone indices from ribs. Figure S4. The linear regressions of max/average diving depth with Cp and Cg for 24 aquatic mammals. [file 12862_2019_1509_MOESM2_ESM.docx]

a.

|  |  |  | *p*-value | | | | |
| --- | --- | --- | --- | --- | --- | --- | --- |
|  | Group.1 | Group.2 | *S* | *P* | *Cg* | *Cc* | *Cp* |
| 1 | Aquatic | Amphibious | 0.01 | 1 | 0.714 | 0.01 | 0.01 |
| 2 | Aquatic | Arboreal | 0.016 | 1 | 1 | 0.01 | 0.01 |
| 3 | Aquatic | Terrestrial | 0.01 | 1 | 1 | 0.01 | 0.01 |
| 4 | Aquatic | Flying | 0.028 | 0.9 | 0.252 | 0.014 | 0.287 |
| 5 | Amphibious | Arboreal | 1 | 1 | 1 | 0.892 | 1 |
| 6 | Amphibious | Terrestrial | 1 | 1 | 0.714 | 1 | 1 |
| 7 | Amphibious | Flying | 1 | 1 | 0.09 | 0.984 | 1 |
| 8 | Arboreal | Terrestrial | 1 | 1 | 1 | 0.222 | 1 |
| 9 | Arboreal | Flying | 1 | 1 | 0.427 | 1 | 1 |
| 10 | Terrestrial | Flying | 0.78 | 1 | 0.288 | 0.65 | 1 |
| *Lambda* | | | 0 | 0 | 0 | 0 | 0.064 |
| *F* | | | 6.384 | 1.565 | 2.875 | 8.207 | 7.739 |
| *P* | | | 0.001 | 0.213 | 0.04 | 0.001 | 0.001 |

b.

|  | Lambda | *F* | *P* (Aquatic vs Terrestrial) |
| --- | --- | --- | --- |
| *Cc* | 0 | 17.81 | 0.006 |
| *Cg* | 0 | 1.062 | 0.431 |
| *Cp* | 0 | 6.713 | 0.031 |
| *P* | 1 | 0.009 | 0.945 |
| *S* | 0.676 | 15.864 | 0.017 |

Table S5. Data from phylogenetically informed ANOVA. Summary of pairwise comparison results of bone quantitative indices in different habitats using the phytools package in R. a. data from rib. b. data from humerus.


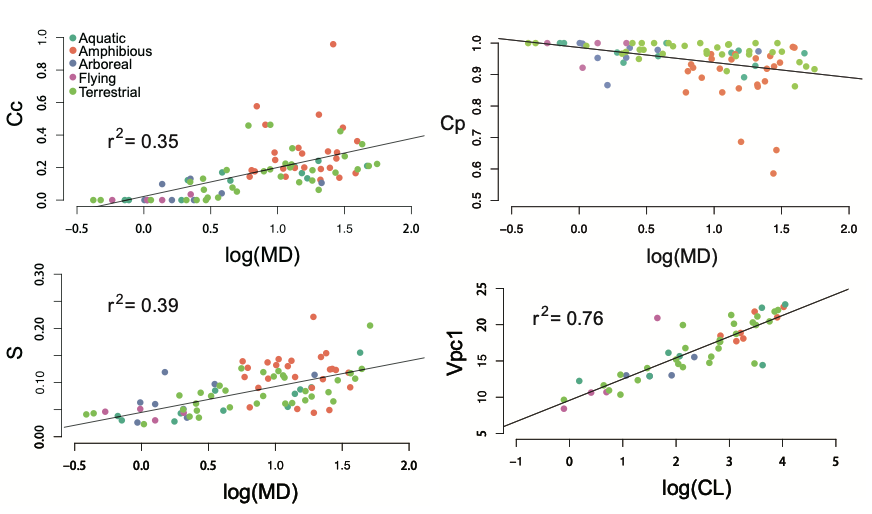


Figure S1. Linear regressions result of *MD* and bone parameter (*S*, *Cc*, *Cp*) from rib; Linear regression result of *CL* and *Vpc1* from vertebrae.

Figure S2. A phylogeny of 24 marine mammals with corresponding bone section and the max diving depth for each species. The section images are *Tursiops truncatus* PL15-0145-28, *Delphinus capensis* PL15-0145-07, *Stenella attenuata* PL15-0145-14, *Sousa chinensis* PL15-0145-16, *Globicephala melas* AMNH 215271, *Pseudorca crassidens* PL15-0145-03, *Lissodelphis borealis* AMNH 31422, *Orcinus orca* AMNH 34261, *Neophocaena asiaeorientalis* PL15-0145-21, *Phocoena phocoena* MHNL 50001046, *Delphinapterus leucas* AMNH 34936, *Monachus monachus* MHNL 50001018, *Inia goeffrensis* AMNH 209101, *Lipotes vexillifer* PL15-0145-18, *Mesoplodon densirostris* AMNH 139931, *Balaenoptera acutorostrata scammoni* PL15-0145-08, *Zalophus californianus* AMNH 63946, *Eumetopias jubatus* AMNH 38400, *Arctocephalus pusillus* AMNH 81701, *Odobenus rosmarus* MHNL 50001014, *Cystophora cristata* AMNH 184659, *Phoca vitulina* MHNL 50001020, *Monachus monachus* MHNL 50001018, *Enhydra lutris* MHNL 50001023.


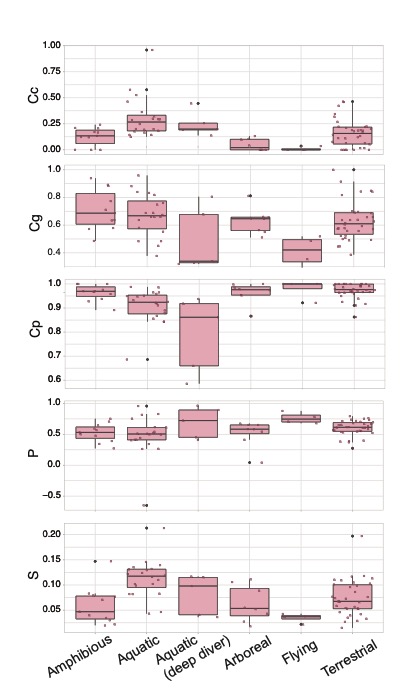


Figure S3. Box plot of six habitat categories for five bone indices from ribs.

Figure S4. The linear regressions of max/average diving depth with Cp and Cg for 24 aquatic mammals.
